# Role of ^18^F-AlF-NOTATATE PET/CT in selecting pediatric neuroblastoma candidates for ^177^Lu-DOTATATE peptide receptor radionuclide therapy

**DOI:** 10.3389/fmed.2025.1615136

**Published:** 2025-08-28

**Authors:** Yuxuan Liu, Yingying Sun, Di Zuo, Han Wang, Fei Zheng, Jingfu Wang, Xiaorong Sun

**Affiliations:** ^1^Department of Nuclear Medicine, Shandong Cancer Hospital and Institute, Shandong First Medical University and Shandong Academy of Medical Sciences, Jinan, China; ^2^Department of Nuclear Medicine, Qingdao Traditional Chinese Medicine Hospital, Qingdao Hiser Hospital Affiliated of Qingdao University, Qingdao, China; ^3^Department of Pediatric Oncology, Shandong Cancer Hospital and Institute, Shandong First Medical University and Shandong Academy of Medical Sciences, Jinan, China

**Keywords:** neuroblastoma, radionuclide therapy, peptide receptor radionuclide therapy, quantitative, therapy response

## Abstract

**Background:**

Neuroblastoma is the most common extracranial solid tumor in children. Peptide receptor radionuclide therapy (PRRT) is a treatment modality with great potential, however, the predictive indicators for its efficacy remain unclear. The aim of the study is to evaluate the prognostic utility of quantitative metrics obtained from ^18^F-AlF-NOTATATE PET/CT at baseline and post-treatment for predicting response in PRRT in pediatric neuroblastoma.

**Methods:**

Patients with high-risk neuroblastoma that was either recurrent or resistant to treatment were prospectively enrolled for one or two cycles of ^177^Lu-PRRT. ^18^F-AlF-NOTATATE PET/CT was performed 1 month before and after PRRT; some patients underwent mid-treatment scans (7 weeks post-cycle). Treatment response was evaluated using a modified approach combining principles from European Organization for Research and Treatment of Cancer (EORTC) criteria and Response Evaluation Criteria In Solid Tumors (RECIST version 1.1) criteria. Lesions were delineated semiautomatically to obtain maximum standardized uptake value (SUV_max_), mean standardized uptake value (SUV_mean_), ratio of tumor SUV_max_ to liver SUV_max_ (SUV_T/L_), ratio of tumor SUV_max_ to spleen SUV_max_ (SUV_T/S_), tumor volume, total lesion activity, and heterogeneity values. Data were analyzed using independent *t*-tests or Mann–Whitney U tests. Receiver operating characteristic curves were used to determine the optimal cut-offs for PET parameters.

**Results:**

Twenty-two patients (13 boys, 9 girls) were included. Baseline PET revealed significantly lower SUV_T/S_, tumor volume, and total lesion activity in non-progressive lesions (*p* < 0.05); SUV_T/S_ predicted efficacy (area under the curve [AUC], 0.588). Interim PET showed significantly lower SUV_max_, SUV_mean_, SUV_T/L_, and SUV_T/S_ in non-progressive lesions (*p* < 0.05); SUV_T/L_ predicted efficacy (AUC, 0.740). The SUV_max_ ratio (interim/baseline) had the highest predictive accuracy, with a cut-off of 1.25 (AUC, 0.796; sensitivity, 73.03%; specificity, 76.92%).

**Conclusion:**

Quantitative baseline and mid-treatment ^18^F-AlF-NOTATATE PET/CT-derived parameters possess value in predicting PRRT response. An interim-to-baseline PET-derived lesion SUV_max_ ratio of ≤1.25 can effectively predict neuroblastoma response to PRRT.

## 1 Introduction

Neuroblastoma is the most prevalent extracranial solid tumor in children, representing 7–8% of all pediatric cancers (1). Approximately 50% of patients are classified as high-risk at the time of diagnosis (2). Despite intensive multimodal treatment, high-risk patients with neuroblastoma experience refractory disease or relapse (3). Peptide receptor radionuclide therapy (PRRT) targets the somatostatin receptor (SSTR) and delivers targeted radiation to SSTR-expressing cells *in vivo* (4). Among PRRT agents, ^177^Lu-DOTATATE has demonstrated significant efficacy and gained regulatory approval in many regions, particularly for SSTR-positive neuroendocrine tumors (NETs) (5, 6). The National Comprehensive Cancer Network (NCCN) (7) has identified PRRT as a therapeutic option for patients with advanced metastatic gastrointestinal, bronchopulmonary, and thymic NETs. Given its success in NETs, there is growing interest in exploring the utility of ^177^Lu-DOTATATE and similar SSTR-targeting radiopharmaceuticals in other SSTR-expressing malignancies, including neuroblastoma. Despite of the increasing use of PRRT for neuroblastoma, data on its efficacy are limited. The disease control rate (DCR) varies across studies, and reliable indicators for predicting treatment responses are lacking. Gains et al. (8) evaluated six children with neuroblastoma who underwent two or three cycles of PRRT, yielding a DCR of 83.33%. However, another study observed no objective responses in 20 children who underwent one to four cycles of PRRT (9). Further, Malcolm et al. (10) reported a DCR of 100% (*n* = 6) for neuroblastoma after four PRRT cycles. These discrepancies may be attributed to the heterogeneity among patient populations, differences in treatment regimens, and assessment criteria. ^68^Ga-DOTATATE positron emission tomography/computed tomography (PET/CT) enables the visualization of tumor SSTR2 expression using radiolabeled somatostatin analogs (11). In comparison to conventional SSTR scintigraphy (e.g., ^111^In-octreotide scanning), ^68^Ga-DOTATATE PET/CT offers superior spatial resolution, higher detection rates for minute lesions, and enhanced quantitative capabilities (12). SSTR PET/CT, represented by ^68^Ga-DOTATATE PET/CT, allows for direct visualization of PRRT targets. Unlike ^18^F-FDG PET/CT, it provides precise molecular-level guidance for PRRT, thereby establishing it as a pivotal instrument in the theranostic paradigm. An in-depth analysis of SSTR PET/CT parameters may assist in identifying PRRT-sensitive patients.

Multiple studies have revealed that baseline ^68^Ga-PET standardized uptake values (SUVs), volumetric parameters, and heterogeneity parameters may aid in predicting the response to PRRT in patients with NETs. Previous studies have demonstrated that the maximum SUV (SUV_max_) can help predict the treatment response and progression-free survival (PFS) (13–17). However, others found no significant correlation between SUV_max_ and the treatment response (18, 19). Ortega et al. (14) demonstrated that an elevated ratio of tumor SUV_max_ to liver SUV (SUV_T/L_) on baseline PET serves as a predictor for extended-progression PFS, whereas Durmo et al. (20) identified a correlation between increased tumor volume (TV) and diminished overall survival. Laudicella et al. (21) and Atkinson et al. (22) found that heterogeneity parameters, viz. skewness, kurtosis, and entropy, can help predict treatment response. However, the use of ^68^Ga-labeled tracers is limited by issues such as low production yield, short half-life, and high cost (23, 24). Compared to ^68^Ga-labeled somatostatin analogues, ^18^F-AlF-NOTATATE offers significant advantages, including a longer half-life, higher production yield, and superior image resolution, positioning it as a promising replacement with enhanced diagnostic performance (25–27). To our knowledge, no study has investigated the potential of ^18^F-AlF-NOTATATE PET/CT in predicting PRRT efficacy in neuroblastoma patients.

Therefore, we aimed to assess the value of SUVs, volumetric parameters, and heterogeneity parameters derived from ^18^F-AlF-NOTATATE PET/CT for predicting the efficacy of PRRT in patients with neuroblastoma, to assist in the clinical screening of patients who may benefit from ^177^Lu-DOTATATE PRRT.

## 2 Methods

### 2.1 Patients

Data were prospectively collected from pediatric patients with high-risk neuroblastoma who were refractory or recurrent and scheduled for PRRT between December 2022 and August 2023. Patients underwent baseline ^18^F-AlF-NOTATATE PET/CT to determine their eligibility. The inclusion criteria were: (1) age range of 0–18 years; (2) neuroblastoma confirmed through histological analysis; (3) failure to achieve complete remission of stage IV recurrent or refractory high-risk neuroblastoma with conventional therapy; (4) primary or metastatic tumors showing greater uptake on [^18^F]AlF-NOTATATE PET/CT than liver uptake; (5) at least 1 month elapsed since the last intravenous chemotherapy, with recovery from hematologic toxicity; (6) stable vital signs and expected survival of no less than 3 months. Criteria for exclusion were: (1) rapid disease progression; (2) prior or ongoing treatment with other somatostatin analogues; and (3) presence of other malignancies requiring active treatment.

Selective interim PET was performed 7 weeks after the first PRRT cycle to explore its prognostic value for clinical outcomes; follow-up ^18^F-AlF-NOTATATE PET/CT was performed 1 month following the final PRRT cycle. The study was authorized by the Ethics Committee of Shandong Cancer Hospital and Institute, and all procedures were conducted in accordance with relevant laws and institutional guidelines. All patients and their guardians provided informed consent, and the privacy rights of all human subjects were respected.

### 2.2 ^18^F-AlF-NOTATATE PET/CT

Patients underwent ^18^F-AlF-NOTATATE PET/CT before PRRT (baseline), 7 weeks after the first cycle of PRRT (interim), and 1 month after the last PRRT (post-treatment). The median time between the initial PET/CT scan and the first treatment cycle was 5 weeks (range: 1–22 weeks). All PET/CT scans were performed on a Siemens Biograph PET/CT scanner (Siemens Medical, Erlangen, Germany). Patients were positioned supine; scans covered the region from the skull vertex to the feet. The median dose of ^18^F-AlF-NOTATATE was 126.2 MBq (range: 107.3–240.1 MBq), with an average uptake time of 88.3 min (range: 49–161 min).

Following the methods of Tirosh et al. (28) and Pauwels et al. (29), ^18^F-AlF-NOTATATE-positive tumor lesions were outlined semiautomatically using MIM software.3.2 (Cleveland, OH, USA). The lesions with non-physiological or higher uptake than the background level in the same region were defined as ^18^F-AlF-NOTATATE-positive lesions (25). The volume of interest (VOI) covering the whole-body PET images was specified. VOIs less than 0.1 mL were automatically omitted. All regions showing physiological or non-pathological ^18^F-AlF-NOTATATE uptake were manually excluded. Additionally, small yet distinct tumor lesions exhibiting reduced ^18^F-AlF-NOTATATE uptake, which were initially neglected during segmentation, were manually delineated using the PET Edge® tool. Ultimately, the scenario containing all ^18^F-AlF-NOTATATE-positive tumor lesions was determined, and the SUV_max_, mean SUV (SUV_mean_), TV (the volume of individual lesions, in mL), and total lesion activity (TLA) were computed automatically. TLA was calculated by multiplying SUV_mean_ of the VOI with its volume. SUV_T/L_ and ratio of tumor SUV_max_ to spleen SUV_max_ (SUV_T/S_) were measured and utilized for analysis.

The heterogeneity of SSTR expression in different parts of the tumor was assessed using segmented three-dimensional TVs. To this end, three different first-order heterogeneity radiomic parameters were evaluated: (1) the coefficient of variation, which was calculated by dividing the standard deviation by the SUV_mean_; (2) skewness, the third standardized moment, a measure of the asymmetry of activity distribution at the tumor site; and (3) kurtosis, the fourth standardized moment, a measure of the ‘tailedness’ of the probability distribution (10). All PET/CT images were qualitatively reviewed by two nuclear medicine physicians at a dedicated workstation; discrepancies were resolved under the guidance of a senior physician.

### 2.3 PRRT

The radiopharmaceutical ^177^Lu-octreotide (^177^Lu-DOTATATE/TOC) was provided by ABX (Advanced Biochemical Compounds GmbH, Germany). The levels of neuron-specific enolase were recorded a day before treatment. Hydration with 0.9% saline solution was initiated 4 h before administration and continued for 24 h. A 5% amino acid solution (comprising 2.5% L-lysine and 2.5% L-arginine) was infused intravenously at a rate of 1 L over 4 h, commencing 30 min prior to the injection of the radioactive tracer, to reduce renal radiation exposure. The radioactive tracer was injected into the peripheral vein for at least 20 min. The administered dose per body weight was 100–200 MBq/kg (median, 160 MBq/kg). The treatment cycles were administered at intervals of 8–12 weeks.

### 2.4 Efficacy evaluation

Treatment efficacy was evaluated 1 month following the last treatment session using ^18^F-AlF-NOTATATE PET/CT. The response evaluation criteria were adapted from the approach described by Laudicella et al. (21), which represent a modification combining principles from the European Organization for Research and Treatment of Cancer (EORTC) criteria (30) and Response Evaluation Criteria In Solid Tumors (RECIST version 1.1) criteria (31). Outcomes were categorized as complete response (CR), partial response (PR), stable disease (SD), or progressive disease (PD). CR was defined as the elimination of all lesions. PR was identified by at least a 25% reduction in lesion size or SUV_max_. SD was characterized by less than a 25% increase or decrease in the size or SUV_max_ of the lesions. PD was defined as at least a 25% increase in the size or SUV_max_ of the lesion. All patients and lesions were categorized into the PD and non-PD groups (CR + PR + PD) based on the treatment efficacy.

### 2.5 Statistical analysis

Statistical analyses were performed using SPSS version 26.0. Quantitative variables are presented as medians with interquartile ranges or as means ± standard deviations, while categorical variables are presented as frequencies and percentages. Independent samples *t*-tests or Mann–Whitney U tests were utilized to evaluate the differences between non-PD and PD groups depending on the parametric nature of the data. The relationship between all variables and treatment response was analyzed through binary logistic regression analysis. A receiver operating characteristic (ROC) curve analysis was conducted to assess specificity and sensitivity, and the area under the curve (AUC) was calculated using Youden’s index; *p*-values less than 0.05 were deemed statistically significant.

## 3 Results

### 3.1 Patient characteristics

Twenty-seven children with neuroblastoma underwent baseline ^18^F-AlF-NOTATATE PET/CT between December 2022 and August 2023. Five patients were excluded due to insufficient tracer uptake at the tumor site on baseline ^18^F-AlF-NOTATATE PET/CT (*n* = 2) and withdrawal from the study (*n* = 3). Twenty-two patients, consisting of 13 boys and 9 girls, with a median age of 6 years (range: 2–17 years), met the eligibility criteria, received treatment, and underwent follow-up. Table 1 presents their clinical and tumor characteristics. The primary tumor site was the retroperitoneum in 95.45% of patients and the mediastinum in one patient. All of these primary tumors had been surgically resected prior to PRRT. All patients had residual bone and bone marrow lesions; only four patients had residual lymph node and soft tissue lesions (1 paravertebral nodule and 1 retroperitoneal nodule). Most patients underwent one of two cycles of PRRT: 5 underwent one cycle, and 17 underwent two cycles. The median administered dose was 3518.7 MBq (range: 1665–7,400 MBq).

**Table 1:** Clinical and tumor characteristics of the patient cohort.

Characteristic	Number (%) of patients or Median (range)
Number	22 (100%)
Age	
Median (years)	6 (2–17)
Sex	
Male	13 (59.09%)
Female	9 (40.91%)
Primary tumor	
Retroperitoneal	21 (95.45%)
Mediastinum	1 (4.55%)
Metastasis	
Bone and bone marrow	22 (100%)
Lymph node	2 (9.09%)
Soft tissue	2 (9.09%)
MYCN status	
Amplified	3 (13.64%)
Not amplified	14 (63.64%)
Unknown	5 (22.73%)
NSE (ng/mL)	18.45 (12.30–51.80)
Treatment before PRRT	
Surgery	22 (100%)
Chemotherapy	22 (100%)
Radiotherapy	13 (59.09%)
Targeted therapy or immunotherapy	8 (36.36%)
Number of PRRT cycles	
1	5 (22.73%)
2	17 (77.27%)
Time between… (days)	
Baseline PET and PRRT	40 (6–151)
Interim PET and PRRT	40 (33–46)
Post-treatment PET and PRRT	46 (32–109)
PRRT cycles	59 (45–119)

PRRT, peptide receptor radionuclide therapy; NSE, neuron-specific enolase.

### 3.2 Efficacy evaluation

**Figure 1:**
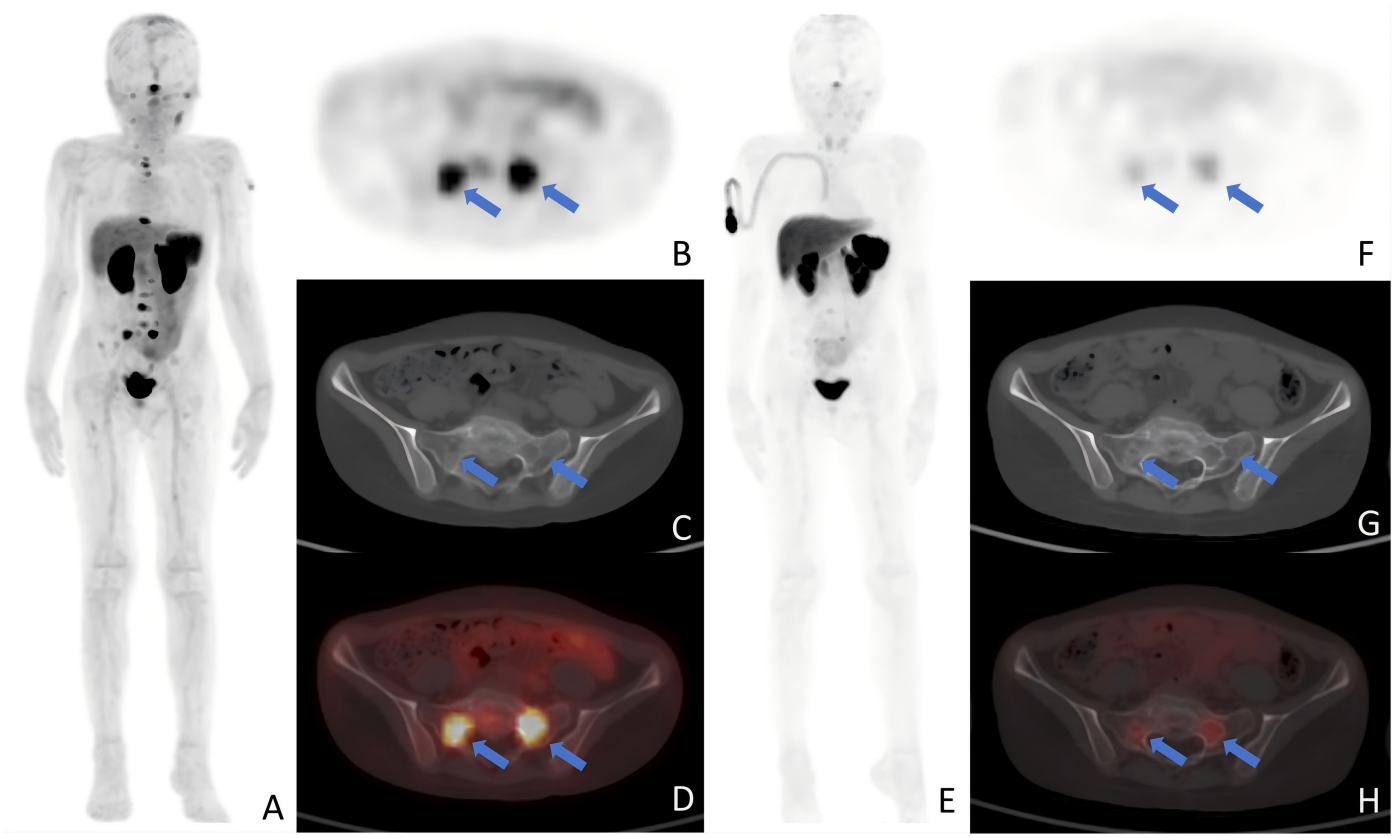
Typical images after PRRT. **(A–D)** A 9-year-old patient with retroperitoneal neuroblastoma prtablesented with a concentration of radioactive uptake in the sacral metastasis (arrows) on baseline PET examination, with an SUV_max_ of 10.07. **(E–H)** One month after two cycles of PRRT, a follow-up PET scan revealed a decrease in the SUV_max_ of the sacral lesion (arrows) to 3.91. Panels show: **(A,E)** MIP; **(B,F)** axial PET; **(C,G)** axial CT bone window; **(D,H)** fused PET/CT SUV_max_, maximum standardized uptake value; PRRT, peptide receptor radionuclide therapy; MIP, maximum intensity projection; PET, positron emission tomography; CT, computed tomography.

One month after the final PRRT session (median, 1; range, 1–4), all patients underwent ^18^F-AlF-NOTATATE PET/CT evaluation, which identified PR, SD, and PD in 4, 8, and 10 patients, respectively; the DCR was 54.55%. Among the patients who received one cycle of PRRT, one exhibited SD, and four demonstrated PD, resulting in a DCR of 20%. For those who received two cycles, four achieved PR, seven had SD, and six experienced PD, with a DCR of 64.71%. Renal toxicity was not observed in any patient; 63.43, 59.09 and 40.91% of patients experienced grade 3–4 anemia, leukopenia, and thrombocytopenia, respectively, but recovered quickly. The median follow-up was 7 months (range: 4–11 months). Baseline ^18^F-AlF-NOTATATE PET/CT revealed a total of 494 lesions across all patients, including 487 bone and bone marrow lesions, 5 lymph node lesions, and 2 soft tissue lesions. In the follow-up qualitative assessment, 139 of 494 lesions were categorized as PD and 355 as non-PD (comprising 227 SD, 115 PR, and 13 CR). A representative image is depicted in Figure 1.

### 3.3 Prediction of treatment efficacy

#### 3.3.1 Baseline PET

**Figure 2:**
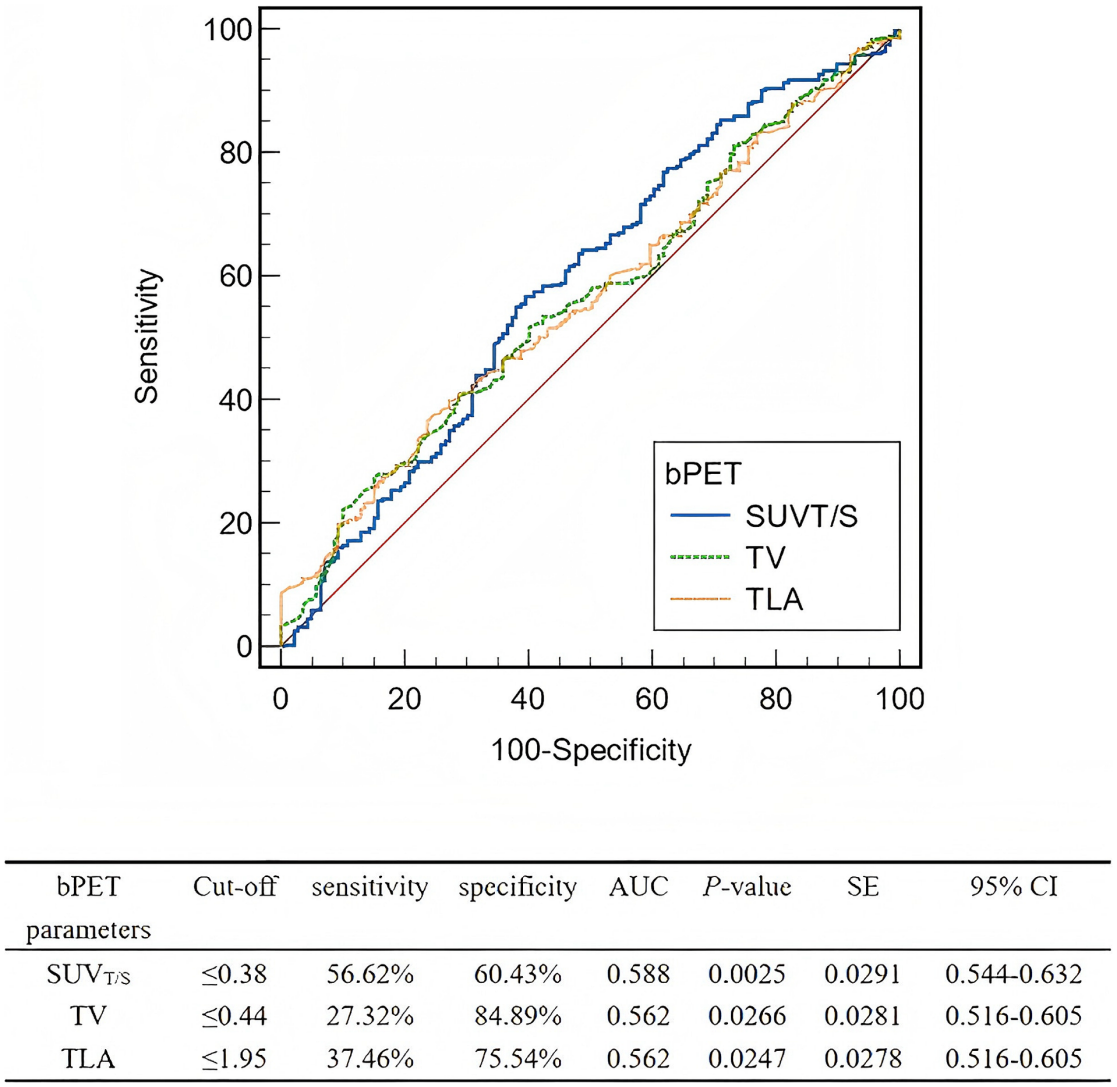
ROCs of the baseline PET parameters for predicting the lesion-based response to PRRT. ROC, Receiver operating characteristic; PRRT, peptide receptor radionuclide therapy; bPET, baseline positron emission tomography; SUV_T/S_, ratio of tumor SUV_max_ to spleen SUV_max_; TV, tumor volume; TLA, total lesion activity; AUC, area under the curve; SE, standard error; CI, confidence interval.

Table 2 depicts the lesion efficacy analysis, where the SUV_T/S_ of non-PD lesions was significantly less than that of PD lesions (*p* = 0.002). However, SUV_max_, SUV_mean_, and SUV_T/L_ did not significantly differ between the non-PD and PD groups (*p* > 0.05). Furthermore, the TV (*p* = 0.032) and TLA (*p* = 0.031) were significantly lower in the non-PD group than in the PD group. The coefficients of variation, skewness, and kurtosis also did not significantly differ between the groups (*p* > 0.05). The AUCs for SUV_T/S_, TV, and TLA in predicting efficacy were similar (Figure 2). The AUC for SUV_T/S_ was 0.588 (optimal cut-off value, 0.38; sensitivity, 56.62%; specificity, 60.43%). The optimal cut-off values for TV and TLA were 0.44 (AUC, 0.562; sensitivity, 27.32%; specificity, 84.89%) and 1.95 (AUC, 0.562; sensitivity, 37.46%; specificity, 75.54%), respectively.

**Table 2:** Lesion-based baseline PET parameters.

Baseline PET parameter	All (*n* = 494)	R (*n* = 355)	NR (*n* = 139)	*p*-value
SUV_max_	4.46 (3.02, 7.01)	4.31 (2.93, 6.88)	4.80 (3.23, 7.29)	0.156
SUV_mean_	3.38 (2.32, 4.77)	3.25 (2.31, 4.63)	3.96 (2.46, 4.93)	0.099
SUV_T/L_	1.14 (0.76, 1.85)	1.07 (0.74, 1.75)	1.24 (0.82, 1.93)	0.141
SUV_T/S_	0.36 (0.22, 0.63)	0.33 (0.20, 0.57)	0.43 (0.24, 0.75)	**0.002**
TV^*^	1.05 (0.51, 2.04)	0.98 (0.37, 1.98)	1.22 (0.63, 2.54)	**0.032**
TLA	3.24 (1.45, 8.08)	3.10 (1.15, 7.53)	3.57 (1.97, 9.28)	**0.031**
CoV	0.16 (0.08, 0.30)	0.17 (0.09, 0.30)	0.15 (0.07, 0.30)	0.120
Skewness	0.40 (−0.02, 0.84)	0.39 (−0.03, 0.83)	0.41 (0.00, 0.88)	0.077
Kurtosis	−0.49 (−0.92, 0.08)	−0.52 (−0.97, 0.09)	−0.42 (−0.80, 0.07)	0.186

R, response; NR, non-response; SUV_max_, maximum standardized uptake value; SUV_mean_; mean standardized uptake value; SUV_T/L_, ratio of tumor SUV_max_ to liver SUV_max_; SUV_T/S_, ratio of tumor SUV_max_ to spleen SUV_max_; TV, tumor volume; TLA, total lesion activity; CoV, coefficient of variation; * TV is defined as the volume of individual lesions, in mL. Data for continuous variables are presented as median (interquartile range). Bold values are statistically significant.

#### 3.3.2 Interim PET

Table 3 shows the results of the lesion efficacy analysis, wherein 191 lesions were observed in the seven patients who underwent interim PET (152 lesions were identified in the non-PD group and 39 lesions in the PD group). The SUV_max_ (*p* = 0.008), SUV_mean_ (*p* = 0.008), SUV_T/L_ (*p* < 0.001), and SUV_T/S_ (*p* = 0.023) values for non-PD lesions were significantly lower than those for PD lesions. However, the TV, TLA, and heterogeneity parameters did not differ significantly between the groups (*p* > 0.05).

**Table 3:** Lesion-based interim PET parameters.

Interim PET Parameter	All (*n* = 191)	R (*n* = 152)	NR (*n* = 39)	*p*-value
SUV_max_	5.14 (3.27, 10.02)	4.62 (3.15, 9.41)	7.84 (4.15, 12.01)	**0.008**
SUV_mean_	4.10 (2.43, 6.16)	3.69 (2.30, 5.51)	4.90 (3.79, 7.27)	**0.008**
SUV_T/L_	1.70 (1.01, 2.87)	1.58 (0.95, 2.60)	3.87 (1.64, 7.49)	**<0.001**
SUV_T/S_	0.28 (0.20, 0.50)	0.25 (0.19, 0.49)	0.40 (0.23, 0.62)	**0.023**
TV^*^	1.07 (0.45, 2.29)	1.05 (0.43, 2.27)	1.10 (0.59, 2.40)	0.518
TLA	3.89 (1.35, 10.75)	3.60 (1.27, 10.30)	5.97 (2.65, 13.47)	0.088
CoV	0.21 (0.16, 0.24)	0.20 (0.15, 0.24)	0.22 (0.17, 0.25)	0.079
Skewness	0.42 (−0.01, 0.84)	0.41 (−0.01, 0.80)	0.52 (0.03, 0.99)	0.276
Kurtosis	−0.56 (−0.93, 0.20)	−0.56 (−0.93, 0.15)	−0.47 (−0.89, 0.73)	0.188

R, response; NR, non-response; SUV_max_, maximum standardized uptake value; SUV_mean_; mean standardized uptake value; SUV_T/L_, ratio of tumor SUV_max_ to liver SUV_max_; SUV_T/S_, ratio of tumor SUV_max_ to spleen SUV_max_; TV, tumor volume; TLA, total lesion activity; CoV, coefficient of variation; * TV is defined as the volume of individual lesions, in mL. Data for continuous variables are presented as median (interquartile range). Bold values are statistically significant.

**Figure 3:**
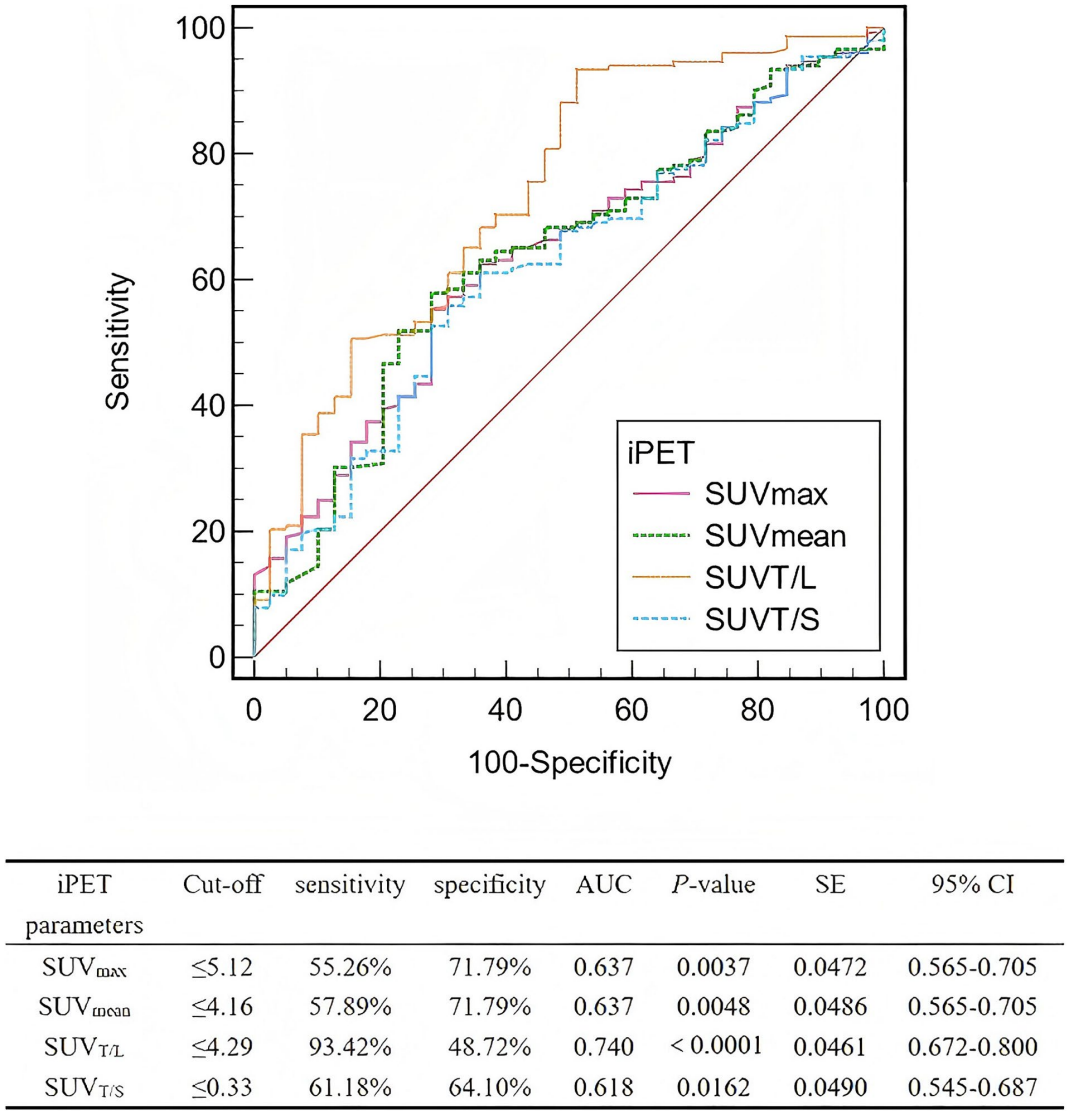
ROCs of the interim PET parameters for predicting the lesion-based response to PRRT. ROC, Receiver operating characteristic; PRRT, peptide receptor radionuclide therapy; iPET, positron emission tomography; SUV_max_, maximum standardized uptake value; SUV_mean_, mean standardized uptake value; SUV_T/L_, ratio of tumor SUV_max_ to liver SUV_max_; SUV_T/S_, ratio of tumor SUV_max_ to spleen SUV_max_; AUC, area under the curve; SE, standard error; CI, confidence interval.

The AUC for SUV_T/L_ was greater than those for SUV_max_, SUV_mean_, and SUV_T/S_ (Figure 3). The optimal cut-off values for SUV_max_, SUV_mean_, SUV_T/L_, and SUV_T/S_ were 5.12 (AUC, 0.637; sensitivity, 55.26%; specificity, 71.79%), 4.16 (AUC, 0.637; sensitivity, 57.89%; specificity, 71.79%), 4.29 (AUC, 0.740; sensitivity, 93.42%; specificity, 48.72%), and 0.33 (AUC, 0.618; sensitivity, 61.18%; specificity, 64.10%), respectively.

#### 3.3.3 Comparison of interim and baseline PET

The ratios of interim-to-baseline SUV_max_, SUV_mean_, SUV_T/L_, and TLA derived from PET in non-PD lesions were significantly lower than those in the PD lesions (all *p* < 0.001; Table 4). However, no significant differences were identified in the interim-to-baseline PET ratios for SUV_T/S_, TV, and heterogeneity parameters between the non-PD and PD groups (*p* > 0.05).

**Table 4:** Lesion-based interim PET/baseline PET ratios.

Interim PET/baseline PET ratios	All (*n* = 191)	R (*n* = 152)	NR (*n* = 39)	*p*-value
SUV_max_	1.16 (1.00, 1.36)	1.12 (0.95, 1.28)	1.46 (1.25, 1.73)	**<0.001**
SUV_mean_	1.13 (0.97, 1.30)	1.10 (0.94, 1.25)	1.36 (1.18, 1.68)	**<0.001**
SUV_T/L_	1.46 (0.78, 2.11)	1.26 (0.75, 1.81)	2.93 (1.27, 3.91)	**<0.001**
SUV_T/S_	0.78 (0.47, 0.99)	0.76 (0.43, 1.00)	0.81 (0.65, 0.95)	0.138
TV^*^	0.99 (0.62, 1.58)	0.92 (0.60, 1.36)	1.21 (0.63, 2.33)	0.102
TLA	1.11 (0.73, 1.76)	1.00 (0.70, 1.48)	1.75 (1.11, 2.97)	**<0.001**
CoV	1.03 (0.88, 1.34)	1.03 (0.90, 1.36)	1.07 (0.83, 1.25)	0.882
Skewness	0.62 (−0.19, 1.22)	0.60 (−0.21, 1.15)	0.68 (−0.15, 2.00)	0.317
Kurtosis	0.65 (−0.27, 1.33)	0.63 (−0.23, 1.30)	0.67 (−0.85, 1.52)	0.780

R, response; NR, non-response; SUV_max_, maximum standardized uptake value; SUV_mean_; mean standardized uptake value; SUV_T/L_, ratio of tumor SUV_max_ to liver SUV_max_; SUV_T/S_, ratio of tumor SUV_max_ to spleen SUV_max_; TV, tumor volume; TLA, total lesion activity; CoV, coefficient of variation; * TV is defined as the volume of individual lesions, in mL. Data for continuous variables are presented as median (interquartile range). Bold values are statistically significant.

**Figure 4:**
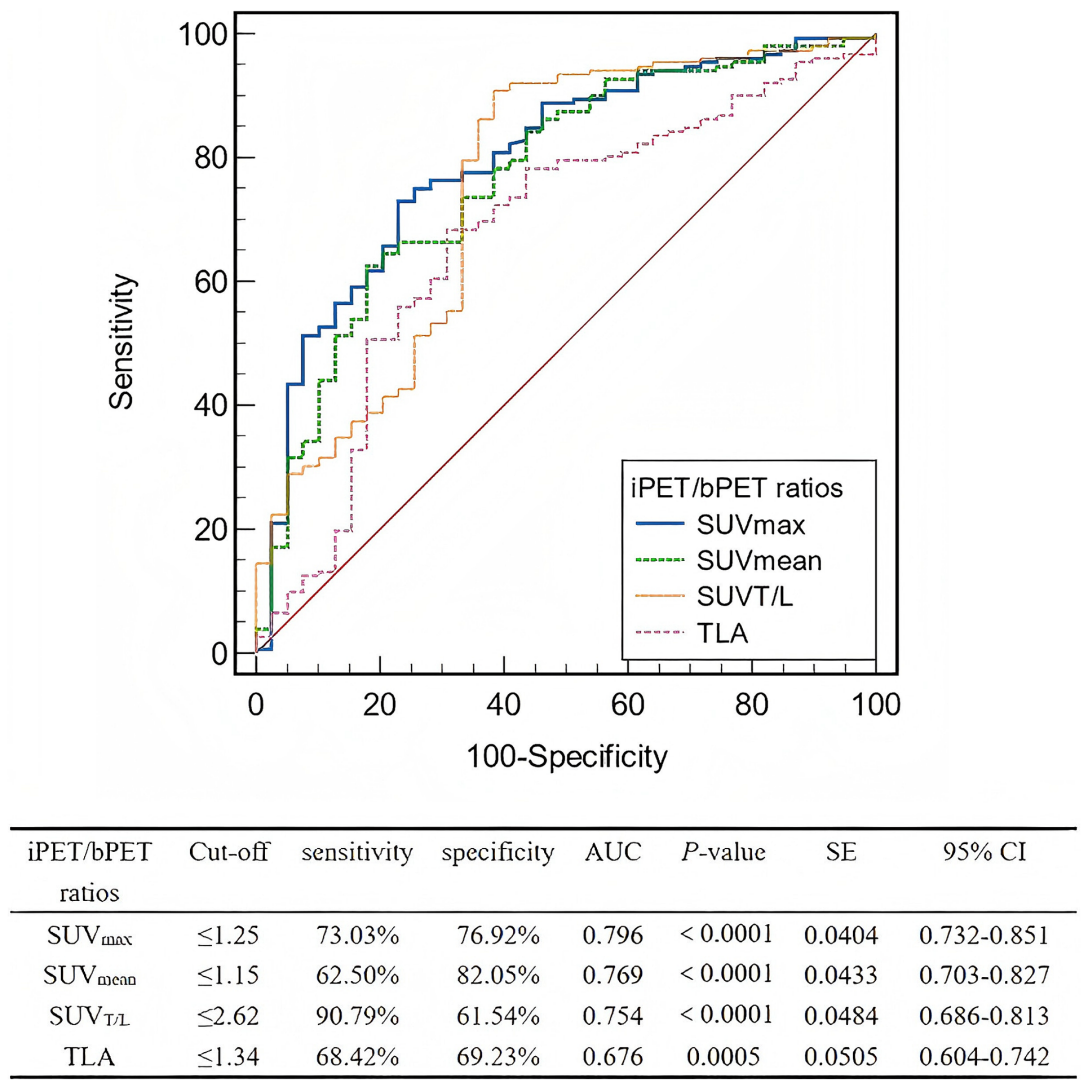
ROC curve analysis of the interim PET/baseline PET ratios for predicting lesion-based response to PRRT. ROC, Receiver operating characteristic; PRRT, peptide receptor radionuclide therapy; iPET, positron emission tomography; bPET, baseline positron emission tomography; SUV_max_, maximum standardized uptake value; SUV_mean_, mean standardized uptake value; SUV_T/L_, ratio of tumor SUV_max_ to liver SUV_max_; TLA, total lesion activity; AUC, area under the curve; SE, standard error; CI, confidence interval.

The AUC for the interim-to-baseline ratio of SUV_max_ was superior to those for SUV_mean_, SUV_T/L_, and TLA (Figure 4). The AUC for the interim-to-baseline ratio of SUV_max_ was 0.796, with an optimal cut-off value of 1.25 (sensitivity, 73.03%; specificity, 76.92%). The AUC for the interim-to-baseline ratio of SUV_mean_ was 0.769, with an optimal cut-off value of 1.15 (sensitivity, 62.50%; specificity, 82.05%). The AUC for the interim-to-baseline ratio of SUV_T/L_ was 0.754, with an optimal cut-off equalling 2.62 (sensitivity, 90.79%; specificity, 61.54%). The AUC for the interim-to-baseline ratio of TLA was 0.676, with an optimal cut-off equalling 1.34 (sensitivity, 68.42%; specificity, 69.23%).

## 4 Discussion

The NCCN recommends PRRT for treating advanced metastatic NETs (7). Although its application in neuroblastoma has gradually increased in recent years, efficacy data are limited; DCRs vary, and reliable efficacy prediction indicators are lacking. Several studies have shown that the SUV_max_ derived from ^68^Ga-DOTATATE/TOC PET/CT can predict PRRT response and PFS (13–17). The results of previous studies further support the predictive value of imaging parameters (14, 20, 22). Compared to ^68^Ga-labled somatostatin analogues, ^18^F-AlF-NOTATATE offers significant advantages, positioning it as a promising replacement with enhanced diagnostic performance. However, currently, studies on the application of ^18^F-AlF-NOTATATE PET/CT in predicting PRRT efficacy in patients with neuroblastoma are lacking. Thus, to our knowledge, ours is the first study to investigate the potential of ^18^F-AlF-NOTATATE PET/CT for forecasting the effectiveness of PRRT in patients with neuroblastoma to assist in the clinical assessment of candidates who may be suitable for PRRT.

We chose ^18^F-AlF-NOTATATE PET/CT to evaluate the effectiveness of PRRT. Although the NCCN guidelines recommend ^123^I-MIBG SPECT/CT as the preferred method for assessing metastatic neuroblastomas (32), these two imaging modalities reflect fundamentally different biological mechanisms. ^123^I-MIBG targets the norepinephrine transporter, whereas ^18^F-AlF-NOTATATE targets SSTR (primarily SSTR2). Given that our study aims to evaluate the efficacy of SSTR-targeted PRRT, selecting a PET tracer that also targets SSTR ensures alignment between the diagnostic assessment and the therapeutic target. This allows for a more direct and precise reflection of the tumor’s response to the treatment. Furthermore, compared to ^123^I-MIBG SPECT/CT, SSTR PET/CT offers superior spatial resolution and higher sensitivity for detecting bone metastases, which further enhances its reliability as a tool for assessing therapeutic efficacy (33). In this study, the DCR of patients treated with one cycle was 20%, and that of patients treated with two cycles was 64.71%, approximating the results of previous studies (8, 9, 34), which reported DCRs of 42.86–100%. Most studies incorporated small sample sizes and retrospective designs, with great inconsistency in clinical questions, inclusion criteria, study design, treatment regimens and survival evaluation. Currently, standardized and effective evaluation criteria for SSTR PET response evaluation are lacking (35, 36).

The reduction in SSTR agonist binding may result from a decrease in SSTRs due to disease progression, treatment effects, or factors like altered perfusion or dedifferentiation (37). Given that the majority of residual lesions were located in the bones and bone marrow, we employed the EORTC criteria for efficacy evaluation. This choice was made because CT/MRI is insufficient for assessing bone marrow lesions, which may not show significant reduction even in the absence of viable tumors (32). Since all patients were children, treatment plans and evaluations required careful consideration. Owing to parental preferences and compassionate care, invasive procedures such as bone marrow aspiration and biopsy could not be performed for all patients (38), complicating the application of INRC criteria (39).

Baseline PET indices such as SUV_max_, SUV_mean_, and SUV_T/L_ did not significantly differ between the non-PD and PD groups, differing from previous NET studies (14). However, SUV_T/S_ and TLA were significantly higher in PD lesions, though their predictive performance was low. The predictive accuracy of SUV_T/S_ and TLA is limited due to intra-tumor and inter-individual heterogeneity, as well as the complex mechanisms of PRRT. In this study, we observed that the lesion TV in the PD group exceeded that in the non-PD group, which is in agreement with previous results (20, 28). The efficacy of ^177^Lu-DOTATATE is influenced by factors such as tumor size due to the limited penetration of the beta emission of ^177^Lu (approximately 0.23 mm) (40). Larger tumors, which often exhibit poor blood supply, are more resistant to radiation (41). Therefore, tumor size and other factors must be considered when planning PRRT for optimal outcomes.

In contrast to previous studies (14, 15, 42), we discovered that the non-PD group showed significantly lower SUV_T/L_ and SUV_max_ of target lesions on mid-treatment PET compared to the PD group. We postulate that this result may be attributable to tumor cell dedifferentiation or the proliferation of SSTR-negative tumor cells during the progression of neuroblastoma. These tumor cells would exhibit lower tracer uptake on SSTR PET/CT, which reflects a loss of the therapeutic target rather than a true tumor response, even as the overall tumor burden increases. Furthermore, neuroblastoma presents with significant biological differences from NET in terms of origin, epidemiology, sites of involvement, and clinical presentation. The potential for selection bias, given that our patient cohort had predominant bone and bone marrow involvement, coupled with insufficient statistical power due to a small sample size, may also be contributing factors to this contrary finding. Laudicella et al. (21) conducted a bone area subgroup analysis in patients with NETs, revealing that SUV_max_ in responders was significantly lower than that in non-responders, consistent with our findings. Moreover, the mid-treatment SUV_mean_ and the mid-to-baseline SUV_mean_ ratio in this study demonstrated better predictive accuracy for treatment efficacy, corroborating with previous results (29, 43). However, Durmo et al. (20) and Werner et al. (44) reported that SUV_mean_ could not reliably predict treatment response or survival in patients undergoing PRRT. SUV_mean_ provides a more comprehensive reflection of tumor lesions than SUV_max_ but is highly influenced by inter-observer variability in tumor delineation. Semi-automatic delineation can mitigate this issue, but SUV_mean_ should not be utilized as the sole parameter for patient screening in PRRT; rather, it can serve as a valuable prognostic factor. This highlights that moving toward normalized or corrected parameters may enhance predictive power. Ilan et al. (45) noted that corrected SUV_max_ values, including tumor-to-blood, tumor-to-spleen, and tumor-to-liver ratios, are more dependable measures than the absolute SUV_max_.

The interplay between cancer cells and the microenvironment during tumorigenesis can greatly influence tumor invasiveness and resistance to treatment (46). Tumors with greater internal heterogeneity often have a worse prognosis. However, no notable differences in heterogeneity parameters were observed between responders and non-responders at baseline or mid-treatment. A key limitation that likely contributes to this finding is that our analysis was confined to first-order, histogram-based texture features (coefficient of variation, skewness, and kurtosis). These metrics describe the statistical distribution of voxel intensities within a tumor but do not capture the spatial relationships or arrangement of those voxels. This approach omits higher-order textural features, such as entropy and those derived from the Gray-Level Co-occurrence Matrix like homogeneity and contrast. Such metrics provide a more sophisticated characterization of textural patterns and have been successfully used in other studies to predict PRRT response (14, 21, 22, 44). It is plausible that while the overall intensity distributions were similar between groups in our cohort, underlying differences in spatial heterogeneity could exist, which our first-order analysis was not designed to detect. Therefore, the lack of significant findings for heterogeneity should be interpreted with caution. Future research incorporating a more comprehensive panel of higher-order radiomic features is warranted to fully explore the predictive value of intratumoral heterogeneity in this setting.

The results of this study demonstrate that the ratios of SUV_max_, SUV_mean_, SUV_T/L_, and TLA between interim and baseline PET scans exhibit favorable predictive performance, with the SUV_max_ ratio showing superior performance among all parameters. These ratios reflect dynamic changes in tumor surface receptor expression before and after treatment, suggesting that early-to-interim PET imaging may serve as a complementary tool for therapeutic efficacy prediction. Furthermore, our findings indicate that dynamic metrics (e.g., multi-timepoint variations) could provide more accurate reflection of tumor response compared to static single-timepoint measurements. However, it is noteworthy that comparative analysis between baseline and interim PET revealed significant increases in single-lesion SUV_max_ at interim assessment in seven patients. This observation should be interpreted with caution. A potential confounding factor is the initial use of ^177^Lu-DOTATOC therapy with relatively lower SSTR2 affinity in all seven cases (47). Despite such influencing factor, it does not diminish our central conclusion. Rather, it reinforces the idea: implementing multi-timepoint PET analysis to evaluate dynamic ratios can enhance the precision of response assessment and provide a stronger basis for personalized treatment.

This study has some limitations that must be considered when interpreting the findings. First, the primary constraint is the study’s single-center design and small sample size, which diminishes the statistical power of our findings and may limit their generalizability. Second, this limitation is compounded by significant heterogeneity within the study cohort. Specifically, while most patients received ^177^Lu-DOTATATE, some were treated with ^177^Lu-DOTATOC, an agent with a relatively lower SSTR2 affinity. As noted in our discussion, this represents a critical confounding factor. In addition, our cohort was predominantly composed of patients with bone and bone marrow metastases, with insufficient representation of soft-tissue lesions. Consequently, the predictive models and conclusions drawn from this study may be most applicable to bone-dominant disease, and their relevance for neuroblastoma patients with primarily soft-tissue involvement remains uncertain. Third, the study lacks a histopathological “gold standard” for validation. Due to ethical and practical challenges associated with performing invasive biopsies in a pediatric population, our efficacy evaluation relied mainly on imaging-based criteria. Therefore, we cannot definitively confirm whether changes in SUV values correspond directly to a change in viable tumor cell count or were influenced by factors such as treatment-related inflammation. Fourth, the limited number of treatment cycles is a constraint, as many patients were unable to complete four treatment cycles due to terminal illness or other barriers. Furthermore, our textural analysis was limited to first-order features, potentially obscuring more complex patterns of spatial heterogeneity that higher-order metrics could have revealed. Lastly, as originally stated, the limited number of treatment cycles and the short follow-up period precluded correlation analyses with long-term clinical outcomes like PFS and overall survival. Future multi-center, prospective studies with standardized treatment and imaging protocols, coupled with long-term follow-up data, are essential to validate and refine our preliminary findings.

## 5 Conclusion

Quantitative parameters based on ^18^F-AlF-NOTATATE PET/CT have great potential in predicting PRRT response in pediatric neuroblastoma. An interim-to-baseline PET lesion SUV_max_ ratio of ≤1.25 can effectively predict the response of that lesion to PRRT, providing a reliable basis for evaluating PRRT efficacy and implementing personalized treatment.

## Data Availability

The original contributions presented in the study are included in the article/supplementary material, further inquiries can be directed to the corresponding authors.
